# A novel transdermal drug delivery system: drug-loaded ROS-responsive ferrocene fibers for effective photoprotective and wound healing activity

**DOI:** 10.1186/s11671-024-04058-w

**Published:** 2024-07-29

**Authors:** Sangwoo Kim, Yoon Kim, Chaehyun Kim, Won Il Choi, Byoung Soo Kim, Jinkee Hong, Hoik Lee, Daekyung Sung

**Affiliations:** 1 Bio-Convergence Materials R&D Division, Center for Bio-Healthcare Materials, Korea Institute of Ceramic Engineering and Technology, 202 Osongsaengmyeong 1-ro, Osong-eup, Heungdeok-gu, Cheongju, Chungbuk 28160 Republic of Korea; 2 Department of Chemical and Biomolecular Engineering, Yonsei University, 50 Yonsei-ro, Seodaemun-gu, Seoul, 03722 Republic of Korea; 3 Advanced Textile R&D Department, Research Institute of Convergence Technology, Korea Institute of Industrial Technology (KITECH), 143 Hanggaulro, Sangnok-gu, Ansan-si, Gyeonggi-do 15588 Republic of Korea; 4 Department of Biosystems & Biomaterials Science and Engineering, College of Agriculture and Life Sciences, Seoul National University, 1 Gwanak-ro, Gwanak-gu, Seoul, 08826 Republic of Korea; 5 Department of Applied Bioengineering, Research Institute for Convergence Science, Seoul National University, Seoul, 08826 Republic of Korea

**Keywords:** Transdermal drug delivery system, Ferrocene, ROS-responsiveness, Fiber, Wound healing, Photo-protectiveness

## Abstract

The present study proposes an innovative transdermal drug delivery system using ferrocene-incorporated fibers to enhance the bioavailability and therapeutic efficacy of ascorbyl tetraisopalmitate. Using electrospinning technology, the authors created ferrocene polymer fibers capable of highly efficient drug encapsulation and controlled release in response to reactive oxygen species commonly found in wound sites. The approach improves upon previous methods significantly by offering higher drug loading capacities and sustained release, directly targeting diseased cells. The results confirm the potential of ferrocene fibers for localized drug delivery, potentially reducing side effects and increasing patient convenience. The method could facilitate the application of bioactive compounds in medical textiles and targeted therapy.

**Supplementary Information:**

The online version contains supplementary material available at 10.1186/s11671-024-04058-w.

## Introduction

Ascorbyl tetraisopalmitate (AT) is a colorless, hydrophobic, and bioactive compound [1, 2] that is one of many derivatives of ascorbic acid, a natural bioactive compound with beneficial effects on the skin, including radical scavenging, inhibition of collagen and melanin production, and wound healing [3–6]. It is resistant to high temperatures, has excellent skin penetration, and is efficiently converted into active vitamin C in the skin, enabling it to perform various physiological functions effectively. However, due to its lipophilic nature, it has limited solubility in water, low bioavailability, and tends to lose activity upon prolonged exposure to light [7, 8]. Such characteristics pose challenges for the development of bio-based products. An effective approach of addressing such issues is the use of drug delivery systems (DDS) [2, 9–11].

In previous work, we have prepared ferrocene-based nanoparticles carrying bioactive substances using nanoprecipitation and other DDS methods [12, 13]. We were able to confirm that ferrocene-containing polymers can be used to encapsulate bioactive substances; however, since much of the research on DDS using AT has focused on encapsulating AT in nanospheres followed by hydrolysis and attachment to membranes to increase drug delivery efficiency [2, 6], we focused on the transdermal drug delivery (TDDS) method, which enables drug delivery through the skin by loading bioactive substances into fiber structures [14]. Therefore, the present study demonstrated a novel approach to DDS research with TDDS using ferrocene fibers, which enables a high degree of drug loading into the polymer structure by electrospinning a ferrocene polymer solution containing AT [15–17]. The method plays an important role in the development of composite systems and carriers because it has advantages such as high drug loading capacity due to high tensile strength, low weight, and large surface area-to-volume ratio, resulting in higher loading efficiency than nanoprecipitation methods [18–20]. There are several strategies for forming fiber structures, such as electrospinning or force spinning [16, 21–23]. Among them, electrospinning is one of the most interesting techniques for implementing effective DDS due to ease of access to polymeric fiber materials [24, 25]. The technique uses electrostatic forces to fabricate fiber structures. In addition, the electrostatic treatment uses a high-voltage electric field to form solid fibers in a polymeric fluid stream (or solution) delivered through a millimeter-scale nozzle [15, 17, 26].

Previous studies have shown that TDDS using common polymers (polyurethane, polyacrylate, polyvinyl alcohol) in the form of fibers are metabolized rapidly in the body. Such systems also have potentially harmful toxic effects on cells when administered at high doses [27, 28]. Therefore, precise and targeted therapeutics are critical for drug delivery systems designed to target diseased cells. Stimuli-responsive formulations represent a new class of programmable delivery systems that can control the release of loaded drugs precisely in response to various intracellular and extracellular biological stimuli (e.g., redox potential, enzymes, and pH) and external stimuli (e.g., ultrasound, light, and temperature) [12, 29–32]. Substances that respond to chemical stimuli, such as reactive oxygen species (ROS), are ideal carriers that exhibit timely drug release patterns in specific physiological environments [29, 33]. In particular, ROS-responsive materials have properties that make them suitable for targeted drug delivery systems in ROS-rich environments, such as wound and inflammatory environments [12, 30]. Ferrocene, one of the representative ROS-stimulatory reactants, is a hydrophobic organic compound that enables reversible self-assembly and controlled drug release [30, 34]. Specifically, under high ROS levels, the ferrocene molecule undergoes oxidation from its hydrophobic neutral state to the hydrophilic ferrocenium cation. Such a transformation facilitates drug release through a transition from hydrophobic to hydrophilic states [26, 34]. With such advantages, fibers composed of ferrocene polymers can control sustained drug release by specific stimuli at the local site, which has the advantages of convenience, durability, and reduced side effects [21, 35].

Fiber constructs that can preserve the bioavailability and bioactivity of AT by encapsulating AT in ROS-responsive ferrocene polymers to facilitate targeted therapy could facilitate the exploration of novel drug delivery systems (DDSs) for effective wound healing [36].

## Methods

### Materials

AT, ferrocenylmethyl methacrylate (FMMA, 95%), methacrylic acid (MA, 99%), tetrahydrofuran (THF; anhydrous, 99.9%), ethyl alcohol (EtOH, 99.5%), an inhibitor removal column, and dimethyl sulfoxide (DMSO-d6) were obtained from Sigma-Aldrich (St. Louis, MO, USA). 2,2-Azobisisobutyronitrile (AIBN, 99%) was purchased from Daejung Chemical (Seoul, Korea). Hydrogen peroxide aqueous solution (H_2_O_2_, 30%) was purchased from Junsei Chemical (Tokyo, Japan), and deionized water (DIW) was obtained from HyClone (Logan, UT, USA). For high-performance liquid chromatography (HPLC), DIW was purchased from Milli-Q^®^ (Molsheim, France), and acetonitrile, isopropanol, and methanol (all HPLC grade) were obtained from Merck^®^ (Darmstadt, Germany). For the *in-situ* antioxidant experiment, the reagents, 2,2-diphenyl-1-picrylhydrazyl (DPPH) and ascorbic acid, (AA) were obtained from Sigma-Aldrich. For in vitro cell incubation, penicillin–streptomycin, trypsin (0.25%), fetal bovine serum (FBS), and Dulbecco’s modified Eagle’s medium (DMEM) were purchased from Gibco (Grand Island, NY, USA). For in vitro experiments, reagents such as 2,7-dichlorodihydrofluorescein diacetate (H2DCFDA) were obtained from Invitrogen (Carlsbad, CA, USA), and phosphate-buffered saline (PBS, pH 7.4) was purchased from HyClone. All solvents were used as received without additional purification.

### Synthesis of ferrocene polymer (FP) and characterization

Ferrocene-containing copolymers were synthesized through radical polymerization following procedures described in previous studies. Before polymerization, MA was purified using an inhibitor removal column. The typical procedure involved dissolving FMMA (0.4 mmol) and MA (2 mmol) in 10 mL of anhydrous THF. AIBN was added (0.12 mmol), and the mixture was degassed by bubbling with argon gas for 10 min. The reaction mixture was stirred at 70 °C for 24 h for the polymerization reaction and then cooled to below 25 °C before being stored at 4 °C until further use. The composition of the resultant copolymers was quantitatively measured using ^1^H NMR by integrating specific peaks: δ = 12.4 (br, 1H, COOH of MA), 4.8 (br, 2H, CO_2_-CH_2_ of FMMA), 4.4–4.1 (br, 9H of FMMA), 2.5 (DMSO‑d_6_), 2.0–1.7 (br, 15H), and 1.1–0.8 (br, 17H). ^1^H NMR spectra were recorded at 400 MHz on a JEOL JNM-ECZ400S/L1 spectrometer (Tokyo, Japan). The solvent used for the recordings was DMSO-d6, and the temperature was maintained at 25 °C. The molecular weights (Mw) and polydispersity indices (PDI = Mw/Mn) were analyzed using gel permeation chromatography (GPC) with an Agilent 1200S/miniDAWN TREOS system and PS calibration (Agilent Technologies, Inc., Santa Clara, CA, USA). THF served as the eluent, flowing at a rate of 1.0 mL/min, and the analysis was conducted at 35 °C [26, 30].

### Fabrication of drug-loaded ferrocene polymer fibers (FPFs) and characterization

Electrospinning experiments were performed using an AT-containing poly (FMMA-r-MA) solution. An electrospinning solution containing 45 wt% poly (FMMA-r-MA) dissolved in EtOH was fixed, and AT concentrations of 20, 45, and 70 wt% with respect to poly (FMMA-r-MA) were prepared. The electrospinning behavior based on the AT composition was investigated. A model NNC-ESR100 electrospinning system (NanoNC, Seoul, Korea) was used for these experiments. It consists of a 30 kV high-voltage generator and a drum-type collector (NNC-DC90H). First, AT-containing poly (FMMA-r-MA) solutions were injected into a plastic syringe fitted with a metallic needle (tip gauge 25). The injection rate was controlled precisely using a syringe pump. The process parameters, including voltage, tip-to-collector distance, and flow rate, were adjusted to 20 kV, 15 cm, and 1 mL/h, respectively. All electrospinning experiments were performed at 25 °C with a relative humidity of approximately 45\–50%. The morphologies of the AT-loaded ferrocene polymer fibers (AT@FPFs) were examined using a field emission electron microscope (FE-SEM, SU8010, Hitachi, Co., Tokyo, Japan). To analyze the response of AT@FPFs to ROS, 0.1% H_2_O_2_ was added to AT@FPFs as an oxidizing agent in aqueous solution (1 mg/mL). To enhance the conductivity of specimens, all SEM specimens were coated with osmium for 60 s using an ion coater (E-1045, Hitachi) before SEM imaging. ImageJ software was used to analyze the SEM images to determine the diameter of the AT@FPFs. The diameter was defined as the length of the longest chord perpendicular to the virtual line drawn along the center of the fiber. The average number and standard deviation (SD) values of the fiber diameter were approximately 1 µm, respectively, for solutions with 20%, 45%, and 70% AT concentration. The chemical composition of the electrospun FPFs was confirmed using Fourier transform infrared (FT-IR) spectroscopy. FT-IR spectra were acquired at 25 °C in the wavenumber range of 4000–450 cm^−1^ using a Perkin Elmer Frontier instrument equipped with a universal attenuated total reflection (UATR) accessory or VERTEX 80 V (Bruker, USA) with KBr pellets. HPLC was performed to verify the loading content and efficiency of AT@FPFs. The chromatographic conditions used for the HPLC experiment were as follows: the injection volume was 20 μL, the detection wavelength was 222 nm, and the mobile phase comprised methanol and isopropanol (25:75 v/v). The eluent flow rate was set at 1 mL/min. All HPLC experiments were performed at 25 °C. The loading content and efficiency were calculated as follows [8]:

1 $$Loading\ content\ \left( \% \right) = \left[ {\frac{{\left( {weight\ of\ fed\ AT - weight\ of\ unloaded\ AT} \right)}}{weight\ of\ FFs}} \right] \times 100$$

2 $$Loading\ efficiency\ \left( \% \right) = \left[ {\frac{{\left( {weight\ of\ fed\ AT - weight\ of\ unloaded\ AT} \right)}}{weight\ of\ fed\ AT}} \right] \times 100$$

### In-situ antioxidant activity of AT@FPFs using the DPPH assay

The antioxidant activity of AT@FPFs was analyzed using the DPPH assay. AT@FPFs were prepared at four AT concentrations (0, 20, 45, and 70 wt%). AA was used as a positive control, and AT in ethanol and 0 wt% AT@FPFs were used as negative controls for comparison with AT@FPFs. To assess the reduction in ROS scavenging efficacy upon exposure to sunlight, AT samples in ethanol and AT@FPFs were prepared and subjected to sunlight exposure for 0, 1, 3, and 7 days. Thereafter, a 0.2 mM DPPH solution was mixed with ethanol and maintained in a dark environment at 4 °C for storage. Afterward, 50 µL of the previously prepared DPPH solution was combined with 150 µL of each sample suspension (AA, AT@FPFs, and AT in EtOH). The negative control group consisted of 50 µL of DPPH solution combined with 150 µL of each sample solvent (DIW or ethanol), and it exhibited little antioxidant activity. Each mixture was maintained in a dark environment at a temperature of 25 °C for 24 h. The absorbance of each mixture was measured at 515 nm using a microplate reader (VICTOR X5; PerkinElmer, Singapore). Antioxidant activity was determined using the following equation [37, 38]:

3 $$Antioxidant\ activity\ \left( \% \right) = \left[ {\frac{{\Delta A_{515}\ of\ control - \Delta A_{515}\ of\ sample}}{{\Delta A_{515}\ of\ control}}} \right] \times 100$$

### In vitro antioxidant activity of AT@FPFs

To evaluate the effectiveness of AT@FPFs at 70 wt% as an intracellular antioxidant, NIH-3T3 fibroblasts were cultured in 24-well plates at a density of 40,000 cells/well for 24 h. ROS generation was induced in NIH-3T3 cells through exposure to H_2_O_2_, an oxidative stress agent. Following the incubation period, PBS was used to wash each 24-well plate and the samples were added to their respective wells. After sample treatment, alterations in ROS levels were measured using subsequent procedures. Initially, NIH-3T3 cells were exposed to suspensions containing AT@FPFs at 70 wt% with various AT concentrations ranging from 0.1 to 100 μg/mL and incubated for 4 h. For comparison, a negative control group without hydrogen peroxide (H_2_O_2_) and a positive control group with H_2_O_2_ were also prepared. Subsequently, after removing any remaining sample solution by washing with PBS (1 mL per 24-well plate), NIH-3T3 cells were exposed to a 10 μM H_2_DCFDA solution, acting as a ROS fluorescence indicator, and allowed to incubate for 30 min in the dark. Finally, the in vitro antioxidant activity of the samples was determined using a microplate reader to measure the fluorescence intensity of DCF at emission and excitation wavelengths of 535 nm and 485 nm, respectively. The intensity of DCF fluorescence is affected by ROS oxidation [12, 37, 38].

### In vitro cell proliferation activity of AT@FPFs

The cell proliferation efficacy of AT@FPFs was assessed by seeding NIH-3T3 cells in a DMEM solution containing 1% penicillin–streptomycin and 10% FBS. Cell proliferation was estimated by measuring cell viability using the Cell Counting Kit-8 (CCK-8) assay. First, the cells were cultured at a density of 7,000 cells/well in 96-well plates. After 24 h of culture, for cell starvation, the cells were cultured in the abovementioned DMEM solution for 4 h. Thereafter, AT@FPFs were added at concentrations ranging from 0.1 to 100 µg/mL and cultured at 37 °C for 24 h. Next, each well of the plate was treated with 1/10 diluted CCK-8 reagent in DMEM solution and cultured for 1 h. The absorbance of formazan produced by the viable cells was measured at 450 nm using a microplate reader (BioTek, Winooski, VT, USA). The percentage of viable cells was calculated using the following equation [39]:

4 $$Cell\ viability\ \left( \% \right) = \left( {\frac{{\Delta A_{450}\ of\ test\ group}}{{\Delta A_{450}\ of\ control\ group}}} \right) \times 100$$

### In vitro wound healing activity of AT@FPFs

To analyze the wound-healing activity of AT@FPFs, a scratch wound-healing assay, which is a widely used method for evaluating cell proliferation and migration, was used. First, 150,000 NIH-3T3 fibroblasts were seeded in each well of a 24-well plate and cultured for 24 h. After the NIH-3T3 cells adhered to the plate, a scratch wound was generated by scraping the cells using a sterile P200 micropipette tip. Cells were treated with AT@FPFs after washing twice with DMEM to eliminate debris. The control group was treated with a DMEM solution. The closure of the cell wound was assessed at different time intervals (0, 6, 12, 24, and 48 h) during a 48-h incubation at 37 °C using a microscope (KI-400, Korea Lab Tech, Korea). The cell wound gaps were calculated using ImageJ software 1.8.0 (National Institutes of Health, Bethesda, MD, USA) [12, 40].

### In vitro cytotoxicity of AT@FPFs

The biocompatibility of AT@FPFs was analyzed by seeding NIH-3T3 cells in DMEM containing 1% penicillin–streptomycin and 10% FBS. Cell viability was evaluated using a CCK-8 assay kit after 24 h of treatment with AT@FPFs. First, cells were seeded at a density of 10,000 cells/well in 96-well plates. After 24 h of seeding, the cells were cultured at 37 °C for 24 h with various concentrations of AT@FPFs (0.1–100 µg/mL). Thereafter, 1/10 diluted CCK-8 reagent in DMEM was added to each well of the plate and incubated for 1 h. The absorbance of formazan produced by viable cells was measured at 450 nm using a microplate reader (BioTek). The percentage of viable cells was calculated using Eq. (4) [41].

### Statistical analysis

The resultant data is presented as mean ± standard deviation. Each experiment was conducted in triplicate. The differences between the experimental groups were compared using the Student's *t*-test. Statistical significance was set at a *p* < 0.05.

## Results and discussion

**Fig. 1:**
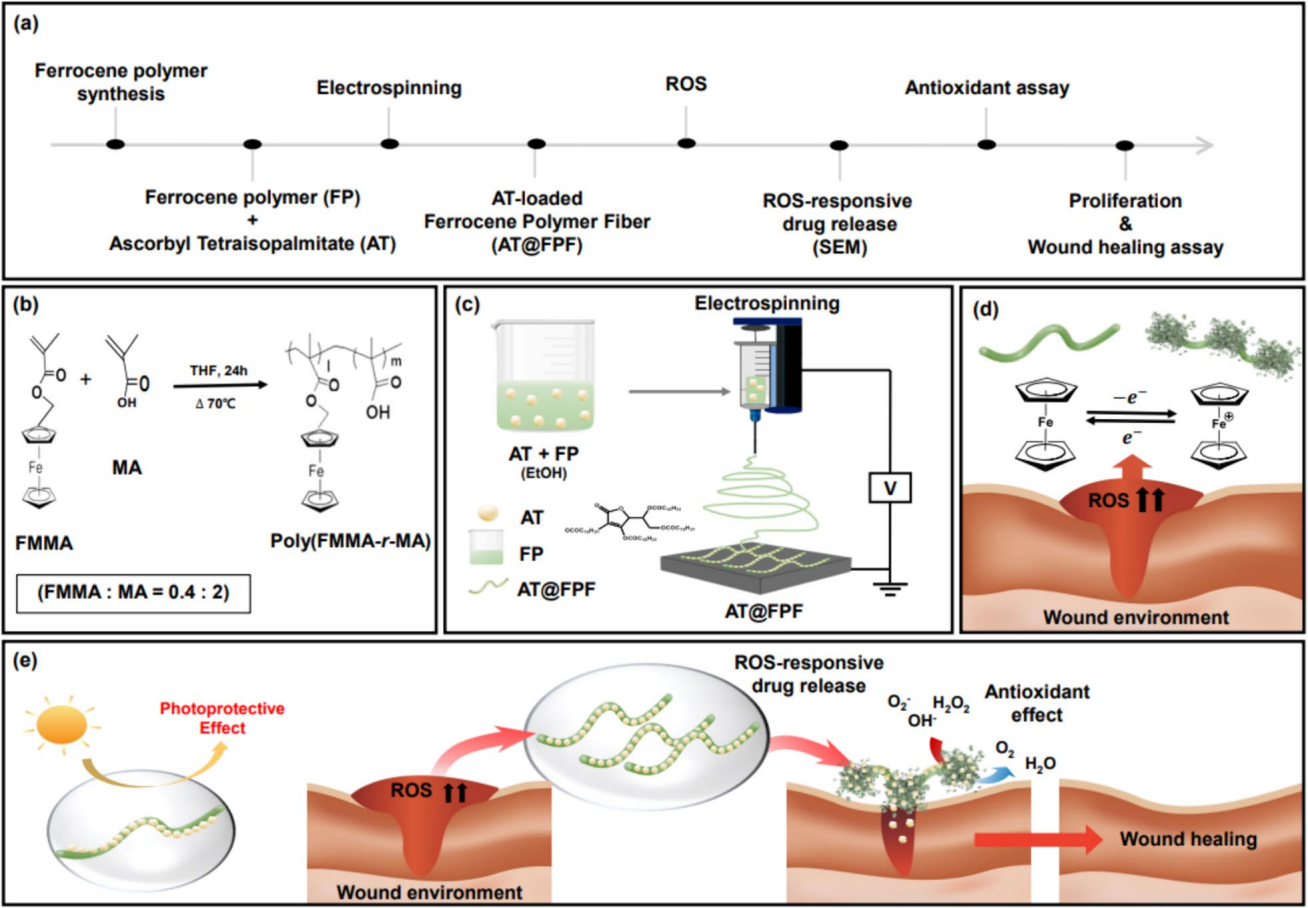
Schematic of **a** The overall experiment schedule, **b** Synthesis of ferrocene polymer (FP), **c** Fabrication of ferrocene polymer fiber (FPF) by electrospinning, **d** ROS-responsive activity of AT@FPF, and **e** Drug-loaded FPF with photoprotective and reactive oxygen species (ROS)-responsive properties for enhanced antioxidant and wound healing effects

**Fig. 2:**
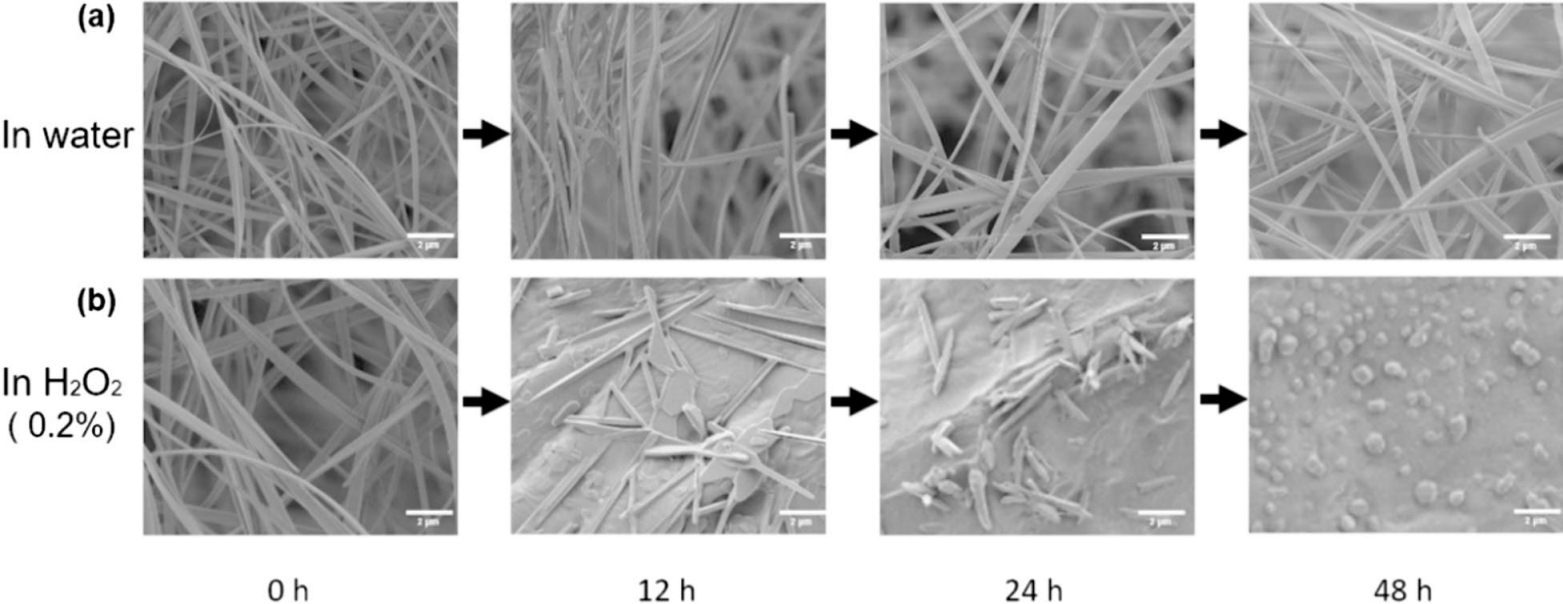
Scanning electrode microscopy (SEM) images showing morphological changes in the ferrocene polymer fiber (FPF) sample after exposure to **a** water and **b** ROS (H_2_O_2_) environments from 0 to 48 h

We initially created a ROS-sensitive amphiphilic ferrocene polymer (FP), poly (FMMA-r-MA), through a straightforward radical polymerization process employing AIBN as the radical initiator [26] This polymer comprised a monomer called FMMA, featuring a hydrophobic Fc segment, and another monomer called MA, which has a hydrophilic COOH group (Fig. 1). To assess the purity of the synthesized FP, we conducted an analysis based on the characteristic monomer peaks found at 5.38 and 6.21 ppm in the ^1^H NMR spectrum, which correspond to the methacrylate protons of the FMMA and MA monomers (Fig. S1). The calculated polymer yield based on the NMR result was determined to be 99.7%, representing the high purity of the polymer. Furthermore, we analyzed the polymers’ Mw and PDI using GPC with polystyrene (PS) calibration, and the findings are detailed in Table S1. The estimated Mw was 5151, with a PDI value of 1.601. These results indicate that the polymerization process effectively yielded the desired FP. By employing this FP, we fabricated an ultrafine fibrous structure via the electrospinning method. Fig. S2 shows the morphology of the electrospun poly (FMMA-r-MA) fibrous structure at 43 w/v%, which exhibits a mixed morphology of beads and fibers. This indicates that the liquid jet at the end of the tip had insufficient surface tension to be stretched to form a uniform fiber structure at a low concentration [42]. Therefore, many unexpected and non-uniform structures with beads were observed. In contrast, when the concentration increased to 45 w/v%, a smooth and fibrous structure without beads was observed (Fig. S3a). A higher concentration enhances the surface tension of the polymer solution, allowing the liquid droplets on the tip to be drawn into a fibrous structure [42]. The heterogeneous structure of the beads and fibers transformed into a fine and uniform fibrous structure as the concentration increased. A subtle increase in the concentration can promote the formation of a more desirable fibrous structure. Therefore, the optimal concentration of poly (FMMA-r-MA) was determined to be 45 w/v% and was used as the base material for subsequent electrospinning experiments involving AT. Fig. S3b–d shows the SEM images of the resulting morphologies of electrospun poly (FMMA-r-MA) with different concentrations of AT. Three different ratios of AT were used: 20%, 45%, and 70% relative to the poly (FMMA-r-MA) content. Remarkably, all samples with different AT ratios exhibited consistent fibrous structures with smooth morphologies through stable electrospinning. This indicated that poly (FMMA-r-MA) can serve as a structural scaffold during electrospinning in the presence of AT. To assess the ROS-responsive properties of AT@FPFs (0 wt%), we observed changes in morphology using H_2_O_2_ as a ROS inducer for 48 h using SEM. The control group (AT@FPFs 0 wt% in water) did not interact with water; thus, there were no morphological changes after 48 h (Fig. 2a). However, in the ROS environment, the ferrocene group (Fe^2+^) was oxidized to a positively charged ferrocenium group (Fe^3+^). The AT@FPFs 0 wt% group was exposed to a 0.2% H_2_O_2_ solution and showed dramatic morphological changes after 48 h (Fig. 2b).

The chemical characteristics of drug and fibers were confirmed by FT-IR, as shown in Fig. S4. The FT-IR spectra revealed distinct peaks characteristic of both the FPFs and AT components, which are also clearly observed in the composite spectrum of AT/FPFs. Specifically, the FPFs exhibited a notable peak at 470 cm^−1^, attributed to the ferrocene conformation [43]. The peaks at 1745 cm^−1^–1804 cm^−1^ are associated with carbonyl groups. The peak at 1745 cm^−1^ typically represents ester carbonyl groups (C=O), often due to the presence of ester linkages [44, 45]. All of these carbonyl peaks are also observed in the AT/FPFs spectrum. In addition, the C–H stretching of CH_2_ is represented by the peaks at 2850 cm^−1^ and 2925 cm^−1^, which were observed predominantly in AT [46]. Considering the chemical structure, this is accepable, and the peaks are expressed more intensively in AT/FP than in FP, indicating the successful loading of AT. Overall, the distinct peak positions offer insights into the chemical functionalities and structures of the AT/FPP, AT, and FPP samples.

To quantify the encapsulation of AT in AT@FPFs, the AT loading content (L.C.; %) and loading efficiencies (L.E.; %) of AT@FPFs were estimated using HPLC at different AT concentrations, and the results are as follows: 20 wt% AT@FPFs: L.C. = 17.11, L.E. = 85.55; 45 wt% AT@FPFs: L.C. = 35.62, L.E. = 79.16; and 70 wt% AT@FPFs: L.C. = 56.51, L.E. = 80.73 (Table S2).

**Fig. 3:**
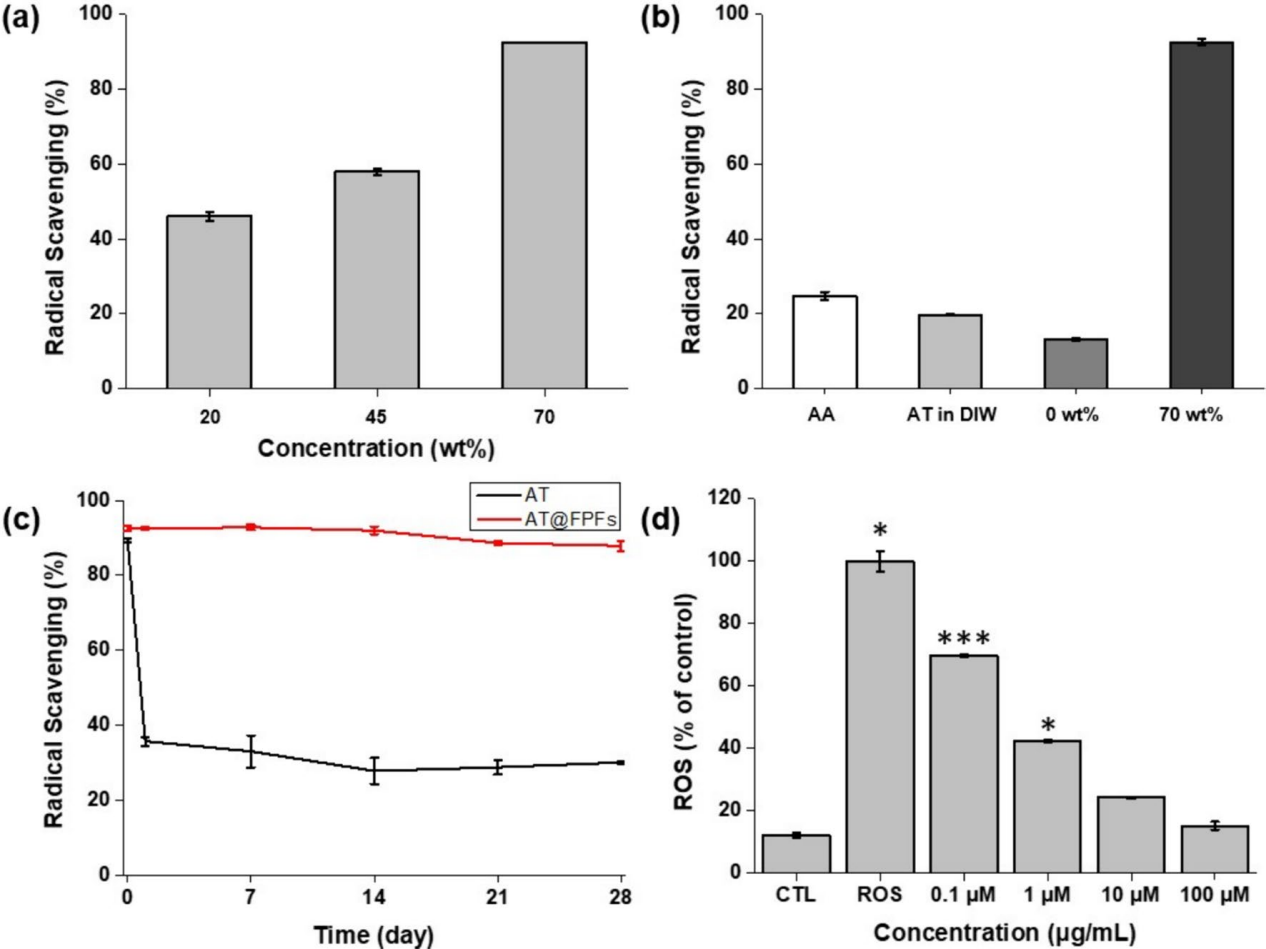
Antioxidant activity results following the DPPH assay. **a** Antioxidant activity of AT@FPFs after changing the loading content from 25 to 70 wt%. **b** Antioxidant activity of AT@FPFs with 0 and 70 wt% AT, compared with that of ascorbic acid (AA) in ethanol (EtOH). **c** Antioxidant activity of 70 wt% AT@FPFs and AT in EtOH after exposure to the sun for 28 days. **d** Results of in vitro assays using the H2DCFDA assay kit. Antioxidation activity of 70 wt% AT@FPFs with AT concentration ranging from 0.1 to 100 μg/mL. The ROS group signifies the highest levels of ROS, whereas the CTL group represents the lowest levels of ROS (*p < 0.05, **p < 0.001)

An *in-situ* DPPH antioxidant activity assay was performed to evaluate the antioxidant activity of AT@FPFs and the other groups [37, 47]. More effective DPPH radical-scavenging activity was observed when the AT@FPFs concentration increased from 20 to 70 wt% (Fig. 3a). Based on the experimental data, we concluded that 70 wt% was the optimal AT loading in AT@FPFs. Moreover, AT in DIW had little effect on the scavenging of DPPH radicals compared with the positive control of AA in DIW. However, AT@FPFs exhibited a more effective DPPH radical-scavenging activity than AA. In addition, in the case of 0 wt% AT@FPFs (without AT), little scavenging of DPPH radicals was observed, suggesting that ferrocene had little effect on the antioxidant activity (Fig. 3b). However, when AT in EtOH was exposed to sunlight for 28 days, its antioxidant properties were dramatically reduced, especially after 24 h (Fig. 3c). These results suggest that AT@FPFs increases antioxidant activity in the water phase and prevents a decrease in efficacy when exposed to light. To measure the in vitro antioxidant activity of AT@FPFs, the H2DCFDA assay was conducted by measuring the H2DCFDA fluorescence after the addition of H_2_O_2_ to mice NIH-3T3 fibroblasts [37, 48]. H_2_O_2_, an oxidative stress agent, leads to the generation of ROS. NIH-3T3 cells treated with H_2_O_2_ were used as negative controls. As expected, the ROS levels of the cells treated with 70 wt% AT@FPFs considerably decreased to approximately 15% as the AT concentration increased from 0.1 to 100 μg/mL (Fig. 3d). These results indicate that AT@FPFs through electrospinning have increased antioxidant efficacy.

**Fig. 4:**
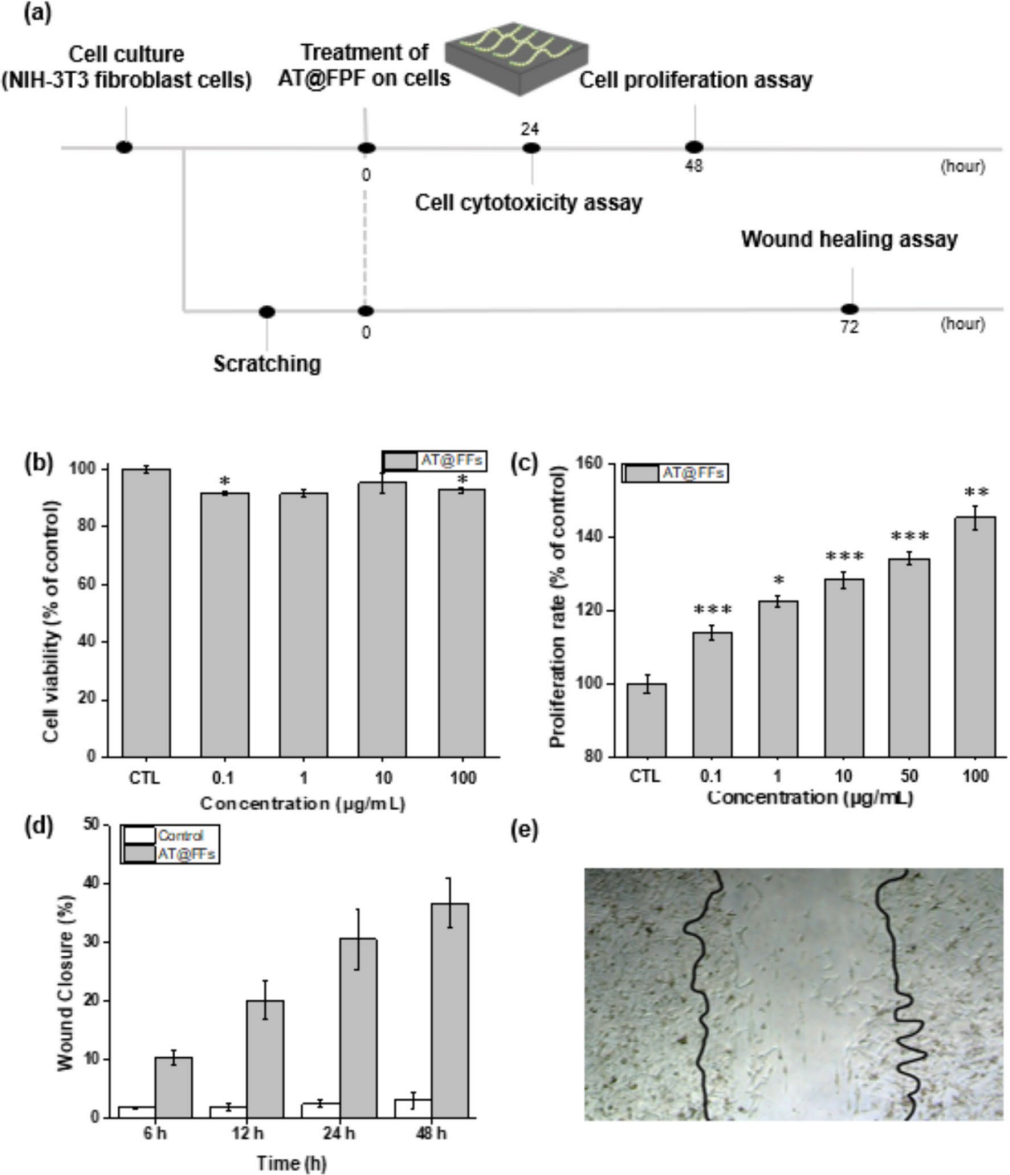
**a** Experimental plan and schedule for AT@FPF in vitro experiments. **b** Cytotoxicity analysis of 70 wt% AT@FPFs at concentrations ranging from 0.1 to 100 μm/mL. **c** Results of in vitro proliferation assays using the CCK-8 assay kit. Cell proliferation activity of 70 wt% AT@FPFs at AT concentrations ranging from 0.1 to 100 mg/mL. **d** Wound closure of NIH-3T3 cells after treatment with AT@FPFs for different periods. **e** Microscopic image of the wound healing activity of AT@FPFs after treatment for 48 h. The black line indicates the initial wound line of NIH-3T3 cells (*p < 0.05, **p < 0.01, and ***p < 0.005)

To verify the biocompatibility of AT@FPFs for wound-healing applications, cytotoxicity was estimated using the CCK-8 assay, which indicates the cell viability of NIH-3T3 cells [41, 49]. No cytotoxic effects were observed when NIH-3T3 cells were exposed to the formulations at concentrations ranging from 0.1 to 100 µg/mL. Cell viability remained unaltered, similar to control untreated cells, irrespective of the tested concentration of AT (Fig. 4a). Therefore, it was confirmed that AT@FPFs are safe for use.

Wound healing is a consequence of cell migration and cell proliferation [50, 51]. Thus, cell proliferation is an important factor in wound healing. In this study, the cell proliferation properties of AT@FPFs were measured using the CCK-8 assay. The cell proliferation activity of AT@FPFs at concentrations ranging from 0.1 to 100 μg/mL increased as AT concentration increased. As expected, the viability levels of NIH-3T3 cells treated with 70 wt% AT@FPFs considerably increased to approximately 40% as AT concentration increased from 0.1 to 100 μg/mL compared with the control groups. Therefore, we consider that 70 wt% AT@FPFs exert a considerable effect on cell proliferation (Fig. 4b). The wound-healing properties of 70 wt% AT@FPFs were estimated using scratch assays on an NIH-3T3 cell monolayer. After 12 h of sample treatment, the degree of wound closure in the untreated cells exhibited little change, whereas the wound closure in the cells treated with AT@FPFs increased by approximately 10% (Fig. 4c). The wound area after 48 h of AT@FPFs treatment significantly increased up to 40% and appeared smaller than the initial wound gap (Fig. 4d). However, the control group, which did not receive any treatment, did not show any changes in wound closure.

## Conclusions

In conclusion, the present study demonstrated that ferrocene polymer fibers effectively encapsulate ascorbyl tetraisopalmitate (AT@FPFs) by electrospinning, achieving high drug loading and stability. This novel transdermal drug delivery system (TDDS) exploits the unique property of ferrocene to respond to ROS, enabling controlled, localized release of AT at targeted sites, such as wounds or areas of inflammation. The mechanism enhances the therapeutic efficacy of AT significantly while minimizing systemic exposure and potential side effects. However, the present study had some limitations, primarily, the scale of production and the long-term stability of the ferrocene fibers under varying environmental conditions, which may affect broader clinical applications. Future studies should focus on optimizing the manufacturing process for larger scale applications and investigating the long-term stability and efficacy of the ferrocene fibers in various clinical settings. In addition, exploring the integration of other bioactive compounds with the ferrocene polymers could expand the utility of this TDDS in the treatment of a wider range of diseases.

### Supplementary Information

Supplementary Material 1.

## Data Availability

Electronic Supplementary Information (ESI) available: [details of any supplementary information available should be included here]. See

## References

[1] Stamford NP. Stability, transdermal penetration, and cutaneous effects of ascorbic acid and its derivatives. J Cosmet Dermatol. 2012;11(4):310–7. 10.1111/jocd.12006. 23174055 10.1111/jocd.12006

[2] Fossa Shirata MM, Maia Campos PMBG. Sunscreens and cosmetic formulations containing ascorbyl tetraisopalmitate and rice peptides for the improvement of skin photoaging: a double-blind, randomized placebo-controlled clinical study. Photochem Photobiol. 2021;97(4):805–15. 10.1111/php.13390. 33529350 10.1111/php.13390

[3] Caritá AC, et al. Vitamin C: one compound, several uses Advances for delivery, efficiency and stability. Nanomed: Nanotechnol Biol Med. 2020;24:102117. 10.1016/j.nano.2019.102117. 10.1016/j.nano.2019.102117 31676375

[4] Duarte TL, Cooke MS, Jones GD. Gene expression profiling reveals new protective roles for vitamin C in human skin cells. Free Radical Biol Med. 2009;46(1):78–87. 10.1016/j.freeradbiomed.2008.09.028. 18973801 10.1016/j.freeradbiomed.2008.09.028

[5] Telang PS. Vitamin C in dermatology. Indian Dermatol Online J. 2013;4(2):143–6. 10.4103/2229-5178.110593. 23741676 10.4103/2229-5178.110593 PMC3673383

[6] Bastianini M, Sisani M, Petracci A. Ascorbyl tetraisopalmitate inclusion into Υ-cyclodextrin and mesoporous SBA-15: preparation, characterization and in vitro release study. Cosmetics. 2017;4(3):21. 10.3390/cosmetics4030021. 10.3390/cosmetics4030021

[7] Brandolini V, et al. Capillary electrophoresis as analytical method for active ingredient determination in cosmetic matrices. Int J Cosmet Sci. 1998;20(1):69–72. 10.1046/j.1467-2494.1998.171737.x. 18505491 10.1046/j.1467-2494.1998.171737.x

[8] Almeida MMD, et al. Determination of tocopheryl acetate and ascorbyl tetraisopalmitate in cosmetic formulations by HPLC. Int J Cosmet Sci. 2009;31(6):445–50. 10.1111/j.1468-2494.2009.00514.x. 19467029 10.1111/j.1468-2494.2009.00514.x

[9] Dochi S, Goldstein D, Microcapsules for effective skin lightening formulations

[10] Fathi-Azarbayjani A, et al. Novel vitamin and gold-loaded nanofiber facial mask for topical delivery. AAPS PharmSciTech. 2010;11:1164–70. 20661676 10.1208/s12249-010-9475-z PMC2974145

[11] Lewińska A, et al. Nanoemulsion stabilized by safe surfactin from *Bacillus**subtilis* as a multifunctional, custom-designed smart delivery system. Pharmaceutics. 2020;12(10):953. 10.3390/pharmaceutics12100953. 33050380 10.3390/pharmaceutics12100953 PMC7601209

[12] Na Y, et al. α-Tocopherol-loaded reactive oxygen species-scavenging ferrocene nanocapsules with high antioxidant efficacy for wound healing. Int J Pharm. 2021;596:120205. 10.1016/j.ijpharm.2021.120205. 33486042 10.1016/j.ijpharm.2021.120205

[13] Na Y, et al. Novel carboxylated ferrocene polymer nanocapsule with high reactive oxygen species sensitivity and on-demand drug release for effective cancer therapy. Colloids Surf B. 2021;200:111566. 10.1016/j.colsurfb.2021.111566 33485085

[14] Tanwar H, Sachdeva R. Transdermal drug delivery system: a review. Int J Pharm Sci Res. 2016;7(6):2274.

[15] Lee H, et al. Electrospun tri-layered zein/PVP-GO/zein nanofiber mats for providing biphasic drug release profiles. Int J Pharm. 2017;531(1):101–7. 10.1016/j.ijpharm.2017.08.081. 28830784 10.1016/j.ijpharm.2017.08.081

[16] Mamidi N, et al. Recent advances in designing fibrous biomaterials for the domain of biomedical, clinical, and environmental applications. ACS Biomater Sci Eng. 2022;8(9):3690–716. 10.1021/acsbiomaterials.2c00786. 36037103 10.1021/acsbiomaterials.2c00786

[17] Li J, Liu Y, Abdelhakim HE. Drug delivery applications of coaxial electrospun nanofibres in cancer therapy. Molecules. 2022;27(6):1803. 10.3390/molecules27061803. 35335167 10.3390/molecules27061803 PMC8952381

[18] Zhang Q, et al. A Biomimetic adhesive and robust Janus patch with anti-oxidative, anti-inflammatory, and anti-bacterial activities for tendon repair. ACS Nano. 2023;17(17):16798–816. 10.1021/acsnano.3c03556. 37622841 10.1021/acsnano.3c03556

[19] García-Valderrama EJ, et al. Engineering and evaluation of forcespun gelatin nanofibers as an isorhamnetin glycosides delivery system. Pharmaceutics. 2022;14(6):1116. 10.3390/pharmaceutics14061116. 35745689 10.3390/pharmaceutics14061116 PMC9229772

[20] Yang Y, et al. Biomimetic, stiff, and adhesive periosteum with osteogenic–angiogenic coupling effect for bone regeneration. Small. 2021;17(14):2006598. 10.1002/smll.202006598. 10.1002/smll.202006598 33705605

[21] Mamidi N, Zuníga AE, Villela-Castrejón J. Engineering and evaluation of forcespun functionalized carbon nano-onions reinforced poly (ε-caprolactone) composite nanofibers for pH-responsive drug release. Mater Sci Eng C. 2020;112:110928. 10.1016/j.msec.2020.110928. 10.1016/j.msec.2020.110928 32409077

[22] Mamidi N, Delgadillo RMV, Castrejón JV. Unconventional and facile production of a stimuli-responsive multifunctional system for simultaneous drug delivery and environmental remediation. Environ Sci Nano. 2021;8(7):2081–97. 10.1039/D1EN00354B. 10.1039/D1EN00354B

[23] Mamidi N, Delgadillo RMV, González-Ortiz A. Engineering of carbon nano-onion bioconjugates for biomedical applications. Mater Sci Eng C. 2021;120:111698. 10.1016/j.msec.2020.111698. 10.1016/j.msec.2020.111698 33545857

[24] Zhang Q, et al. Advanced technology-driven therapeutic interventions for prevention of tendon adhesion: design, intrinsic and extrinsic factor considerations. Acta Biomater. 2021;124:15–32. 10.1016/j.actbio.2021.01.027. 33508510 10.1016/j.actbio.2021.01.027

[25] Zhang Q, et al. Electrospun polymeric micro/nanofibrous scaffolds for long-term drug release and their biomedical applications. Drug Discov Today. 2017;22(9):1351–66. 10.1016/j.drudis.2017.05.007. 28552498 10.1016/j.drudis.2017.05.007

[26] Lee H, et al. Electrospinning/electrospray of ferrocene containing copolymers to fabricate ROS-responsive particles and fibers. Polymers. 2020;12(11):2520. 10.3390/polym12112520. 33138105 10.3390/polym12112520 PMC7694134

[27] Gencturk A, et al. Polyurethane/hydroxypropyl cellulose electrospun nanofiber mats as potential transdermal drug delivery system: characterization studies and in vitro assays. Artif Cells Nanomed Biotechnol. 2017;45(3):655–64. 10.3109/21691401.2016.1173047. 27103498 10.3109/21691401.2016.1173047

[28] Shi Y, et al. A novel transdermal drug delivery system based on self-adhesive Janus nanofibrous film with high breathability and monodirectional water-penetration. J Biomater Sci Polym Ed. 2014;25(7):713–28. 10.1080/09205063.2014.897596. 24641249 10.1080/09205063.2014.897596

[29] Huang Y, et al. Facile fabrication of oxidation-responsive polymeric nanoparticles for effective anticancer drug delivery. Mol Pharm. 2018;16(1):49–59. 10.1021/acs.molpharmaceut.8b00634. 30485109 10.1021/acs.molpharmaceut.8b00634

[30] Na Y, et al. Reactive oxygen species (ROS)-responsive ferrocene-polymer-based nanoparticles for controlled release of drugs. J Mater Chem B. 2020;8(9):1906–13. 10.1039/c9tb02533b. 32043093 10.1039/c9tb02533b

[31] Zhang Q, et al. Shedding light on 3D printing: printing photo-crosslinkable constructs for tissue engineering. Biomaterials. 2022;286:121566. 10.1016/j.biomaterials.2022.121566. 35633590 10.1016/j.biomaterials.2022.121566

[32] Wang W, Xu C, Yoo JW. Advanced technologies for biomedical applications by emerging researchers in Asia-Pacific. Bioeng Transl Med. 2023. 10.1002/btm2.10621. 38023727 10.1002/btm2.10621 PMC10658477

[33] Sobotta FH, et al. Oxidation-responsive micelles by a one-pot polymerization-induced self-assembly approach. Polym Chem. 2018;9(13):1593–602. 10.1039/C7PY01859B. 10.1039/C7PY01859B

[34] Saravanakumar G, Kim J, Kim WJ. Reactive-oxygen-species-responsive drug delivery systems: promises and challenges. Adv Sci. 2017;4(1):1600124. 10.1002/advs.201600124. 10.1002/advs.201600124 PMC5238745 28105390

[35] Zhang Q, et al. An anti-bacterial and anti-cancer fibrous membrane with multiple therapeutic effects for prevention of pancreatic cancer recurrence. Biomater Adv. 2022;137:212831. 10.1016/j.bioadv.2022.212831. 35929264 10.1016/j.bioadv.2022.212831

[36] Zhang Q, et al. Micro-and nano-environment dual-modulated anti-tendon adhesion barrier membranes. Mater Des. 2022;219:110737. 10.1016/j.matdes.2022.110737. 10.1016/j.matdes.2022.110737

[37] Kim S, et al. Facile fabrication of α-bisabolol nanoparticles with improved antioxidant and antibacterial effects. Antioxidants. 2023;12(1):207. 10.3390/antiox12010207. 36671070 10.3390/antiox12010207 PMC9854552

[38] Apak R, et al. Methods of measurement and evaluation of natural antioxidant capacity/activity (IUPAC technical report). Pure Appl Chem. 2013;85(5):957–98. 10.1351/PAC-REP-12-07-15. 10.1351/PAC-REP-12-07-15

[39] Wang C, et al. Long non-coding RNA MALAT1 promotes cholangiocarcinoma cell proliferation and invasion by activating PI3K/Akt pathway. Neoplasma. 2017;64(5):725–31. 10.4149/neo_2017_510. 28592124 10.4149/neo_2017_510

[40] Lee JE, Boo YC. Combination of glycinamide and ascorbic acid synergistically promotes collagen production and wound healing in human dermal fibroblasts. Biomedicines. 2022;10(5):1029. 10.3390/biomedicines10051029. 35625765 10.3390/biomedicines10051029 PMC9138459

[41] Cai L, et al. Comparison of cytotoxicity evaluation of anticancer drugs between real-time cell analysis and CCK-8 method. ACS Omega. 2019;4(7):12036–42. 10.1021/acsomega.9b01142. 31460316 10.1021/acsomega.9b01142 PMC6682106

[42] Lee H, et al. Readily functionalizable and stabilizable polymeric particles with controlled size and morphology by electrospray. Sci Rep. 2018;8(1):15725. 10.1038/s41598-018-34124-0. 30356115 10.1038/s41598-018-34124-0 PMC6200772

[43] Mohammadi N, et al. Differentiation of ferrocene D5d and D5h conformers using IR spectroscopy. J Organomet Chem. 2012;713:51–9. 10.1016/j.jorganchem.2012.04.009. 10.1016/j.jorganchem.2012.04.009

[44] Fumoto E, et al. Determination of carbonyl functional groups in lignin-derived fraction using infrared spectroscopy. Fuel. 2022;318:123530. 10.1016/j.fuel.2022.123530. 10.1016/j.fuel.2022.123530

[45] Segalina A, et al. Cocrystals of nitrofurantoin: how coformers can modify its solubility and permeability across intestinal cell monolayers. Cryst Growth Des. 2022;22(5):3090–106. 10.1021/acs.cgd.2c00007. 10.1021/acs.cgd.2c00007

[46] Yadav MG, et al. Production of 6-O-l-ascorbyl palmitate by immobilized Candida antarctica lipase B. Appl Biochem Biotechnol. 2018;184:1168–86. 28971362 10.1007/s12010-017-2610-5

[47] Yu S, et al. Highly water-dispersed and stable deinoxanthin nanocapsule for effective antioxidant and anti-inflammatory activity. Int J Nanomed. 2023. 10.2147/IJN.S401808. 10.2147/IJN.S401808 PMC10423574 37581101

[48] Yang H, et al. Facile solvent-free preparation of antioxidant idebenone-loaded nanoparticles for efficient wound healing. Pharmaceutics. 2022;14(3):521. 10.3390/pharmaceutics14030521. 35335897 10.3390/pharmaceutics14030521 PMC8951630

[49] Huang X, et al. The Raman deuterium isotope probing method as a new assay for evaluating the cytotoxicity level of the GSK2334470 to the MCF-7 cells. J Raman Spectrosc. 2023;54(3):269–77. 10.1002/jrs.6489. 10.1002/jrs.6489

[50] Patel GK. The role of nutrition in the management of lower extremity wounds. Int J Low Extrem Wounds. 2005;4(1):12–22. 10.1177/1534734605274574. 15860449 10.1177/1534734605274574

[51] Demling RH, Nutrition, anabolism, and the wound healing process: an overview. Eplasty; 2009;9. PMC2642618 19274069

