# Supplementary material for: A novel transdermal drug delivery system: drug-loaded ROS-responsive ferrocene fibers for effective photoprotective and wound healing activity
Source: Discov Nano. 2024 Jul 29;19(1):119. doi: 10.1186/s11671-024-04058-w (PMC11286613; doi:10.1186/s11671-024-04058-w)
Supplement: Supplementary file 1 — Supplementary Material 1. [file 11671_2024_4058_MOESM1_ESM.docx]

**Journal name: Discover Nano**

**Supplementary Information**

**A novel transdermal drug delivery system: drug loaded ROS responsive ferrocene fibers for effective photoprotective and wound healing activity**

Sangwoo Kim ^a,b^, Yoon Kim ^c^, Chaehyun Kim ^a,d^, Won Il Choi ^a^, Byoung Soo Kim ^a^, Jinkee Hong ^b^, Hoik Lee ^c,*^, and Daekyung Sung ^a,*^

^a^Center for Bio-Healthcare Materials, Bio-Convergence Materials R&D Division, Korea Institute of Ceramic Engineering and Technology, 202 Osongsaengmyeong 1-ro, Osong-eup, Heungdeok-gu, Cheongju,
^b^Department of Chemical and Biomolecular Engineering, Yonsei University, 50 Yonsei-ro, Seodaemun-gu, Seoul 03722, Republic of Korea

^c^Advanced Textile R&D Department, Research Institute of Convergence Technology, Korea Institute of Industrial Technology (KITECH), 143 Hanggaulro, Sangnok-gu, Ansan-si, Gyeonggi-do 15588, Republic of Korea

^d^Department of Applied Bioengineering, Research Institute for Convergence Science, Seoul National University, Seoul 08826, Republic of Korea

* Corresponding authors:

Hoik Lee (hoik@kitech.re.kr)

Daekyung Sung (dksung@kicet.re.kr)

S.K., Y.K., and C.K. contributed equally to this work.

**1. Supplementary Figures and Tables**

**
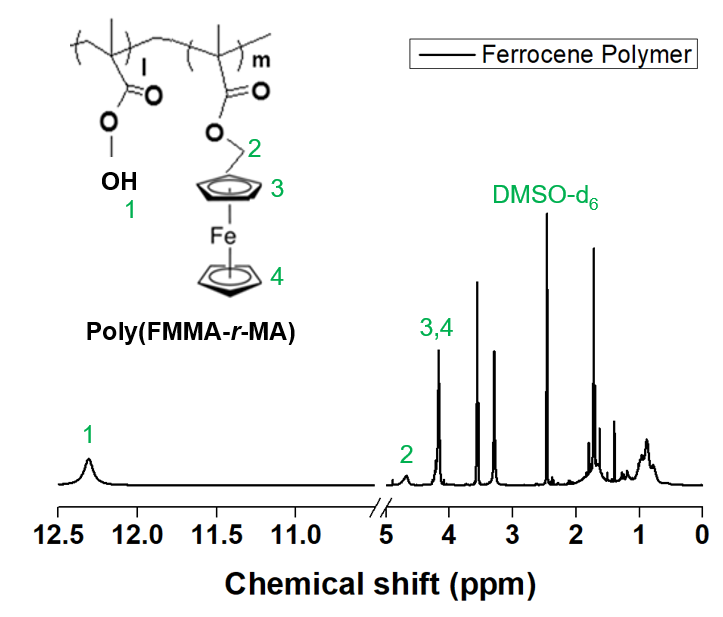
**

**Figure S1**. ^1^H-NMR spectra of the synthesized ferrocene polymers with chemical structures and peak assignments. The proton chemical shifts are expressed in parts per million (ppm) as follows: δ = (1) 12.4 (br, 1H, COOH of MA), (2) 4.8 (br, 2H, CO_2_-CH_2_ of FMMA), (3,4) 4.4–4.1 (br, 9H of FMMA), 2.5 (DMSO‑d6), 2.0–1.7 (br, 15H), and 1.1–0.8 (br, 17H).


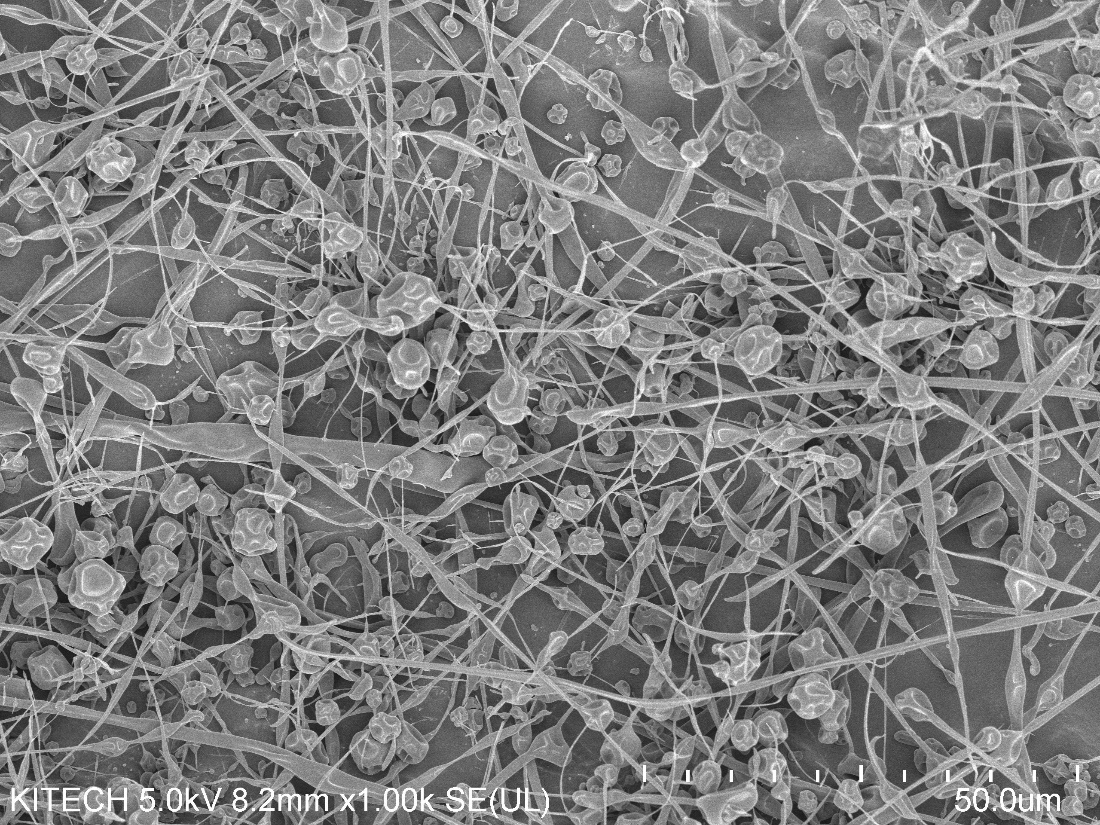


**50 μm**

**Figure S2**. Scanning electron microscopy image of the electrospun fiber with 43 w/v% of poly(FMMA-*r*-MA).

**Figure S3**. Scanning electron microscopy images of the electrospun fibers with (a) 0 wt%, (b) 20 wt%, (c) 45 wt%, and (d) 70 wt% AT relative to the poly(FMMA-r-MA) content.


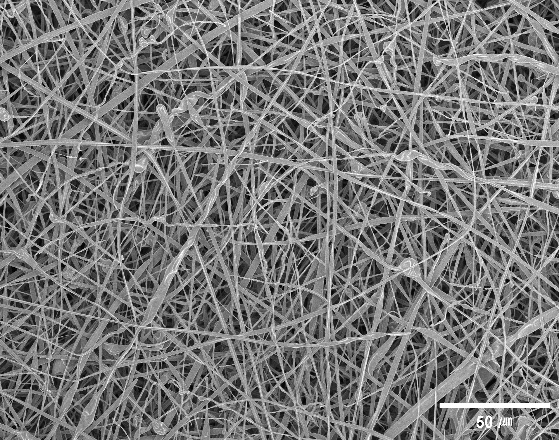

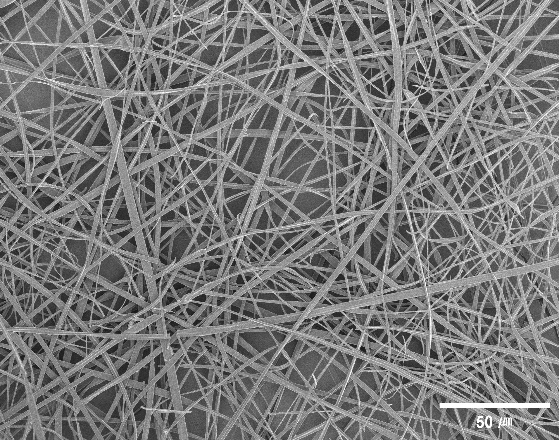

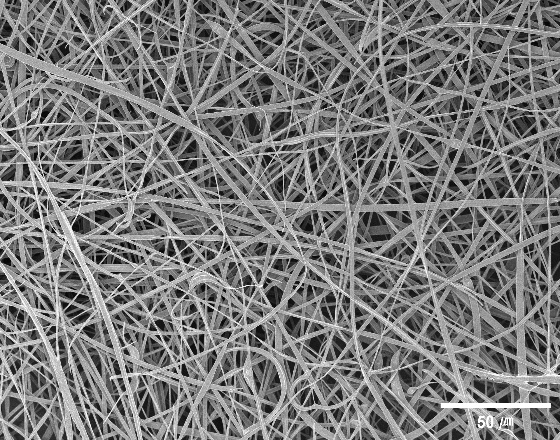

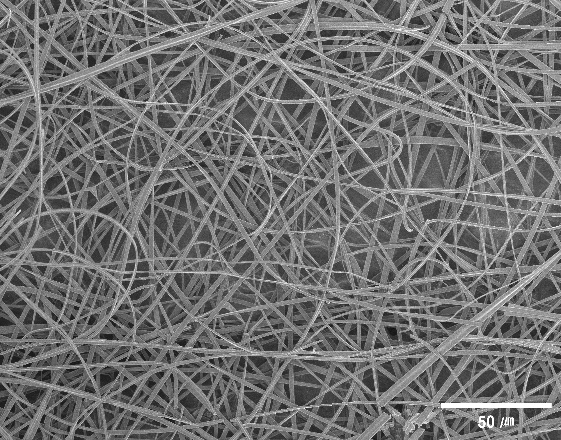


**(a)**

**(b)**

**(c)**

**(d)**

**
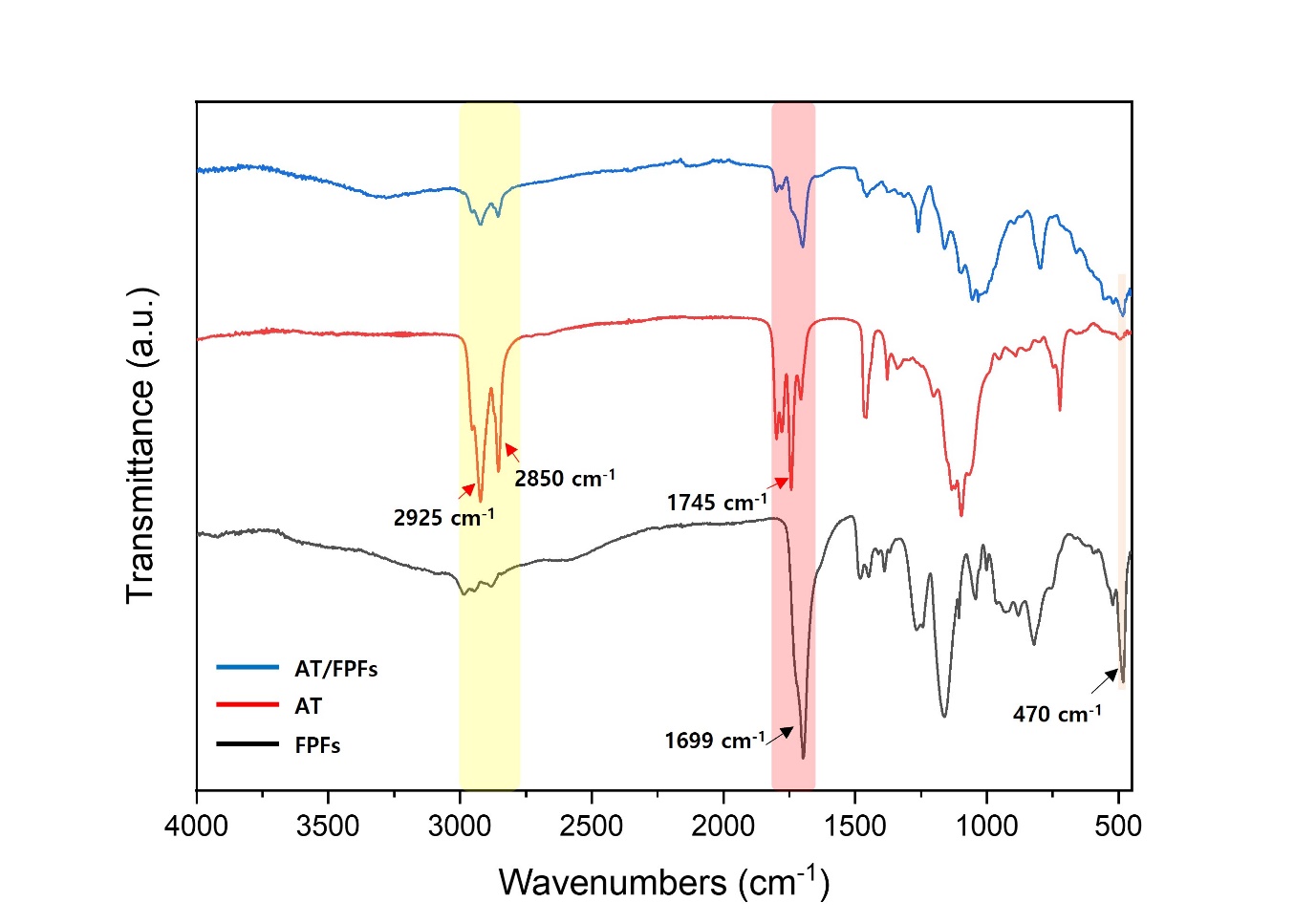
**

**Figure S4**. FT-IR spectra of FPFs (black trace), AT (red trace) and AT/FPFs.

**Table S1**. Monomer feed and ferrocene polymer compositions along with average molecular weight and polydispersity index of the synthesized ferrocene polymers

| **Name** | **Monomer feed (mol%)** | | **Polymer feed (mol%)** | | **Conversion**  **(%)** | **M_n_**  **(g/mol)** | **M_w_**  **(g/mol)** | **D_m_** |  |
| --- | --- | --- | --- | --- | --- | --- | --- | --- | --- |
|  | **FMMA** | **MA** | **FMMA** | **MA** | 99.77 | 3,217 | 5,150 | 1.601 | |
| Ferrocene polymer | 16.7 | 83.3 | 16.0 | 84.0 |  |  |  |  |  |

**Table S2.** HPLC results.

| **AT@FFs** | **Loading contents (L.C.)** | **Loading efficiency (L.E.)** |
| --- | --- | --- |
| **20 wt%** | 17.11 | 85.55% |
| **45 wt%** | 35.62 | 79.16% |
| **70 wt%** | 56.51 | 80.73% |
